# Glycolytic competence in gastric adenocarcinomas negatively impacts survival outcomes of patients treated with salvage paclitaxel-ramucirumab

**DOI:** 10.1007/s10120-020-01078-0

**Published:** 2020-05-05

**Authors:** Annamaria Ruzzo, Francesco Graziano, Irene Bagaloni, Maria Di Bartolomeo, Michele Prisciandaro, Giuseppe Aprile, Elena Ongaro, Bruno Vincenzi, Giuseppe Perrone, Daniele Santini, Lorenzo Fornaro, Caterina Vivaldi, Gianluca Tomasello, Fotios Loupakis, Sara Lonardi, Matteo Fassan, Michele Valmasoni, Donatella Sarti, Paola Lorenzini, Vincenzo Catalano, Renato Bisonni, Michela Del Prete, Guido Collina, Mauro Magnani

**Affiliations:** 1 Department of Biomolecular Sciences (DiSB), University of Urbino “Carlo Bo”, Via Arco d’Augusto, 2, 61032 Fano, PU Italy; 2 Department of Onco-Hematology, Division of Oncology, Azienda Ospedaliera “Ospedali Riuniti Marche Nord”, 61122 Pesaro, Italy; 3 Oncologia Medica GI Fondazione IRCCS Istituto Tumori Milano, Milan, Italy; 4 Department of Medical Oncology, San Bortolo General Hospital, AULSS8 Berica, Vicenza, Italy; 5 Department of Oncology, University and General Hospital, Udine, Italy; 6 University Campus Bio-Medico, Rome, Italy; 7 Azienda Ospedaliero-Universitaria Pisana, Pisa, Italy; 8 S. C. Oncologia, ASST Hospital of Cremona, Cremona, Italy; 9 Department of Oncology, Veneto Institute of Oncology IOV – IRCCS, Padua, Italy; 10 Surgical Pathology Unit, Department of Medicine (DIMED), University of Padua, Padua, Italy; 11 Clinica Chirurgica 3, Department of Surgical, Oncological and Gastroenterological Sciences (DISCOG), University of Padua, Padua, Italy; 12 Medical Oncology Unit, Hospital of Fermo, Fermo, Italy; 13 Area vasta 5, Ospedale “C. e G. Mazzoni” Ascoli Piceno, Ascoli Piceno, Italy

**Keywords:** Glycolysis, Warburg effect, Ramucirumab, Paclitaxel, Angiogenesis

## Abstract

**Introduction:**

For energy production, cancer cells maintain a high rate of glycolysis instead of oxidative phosphorylation converting glucose into lactic acid. This metabolic shift is useful to survive in unfavorable microenvironments. We investigated whether a positive glycolytic profile (PGP) in gastric adenocarcinomas may be associated with unfavorable outcomes under an anticancer systemic therapy, including the anti-angiogenic ramucirumab.

**Materials and methods:**

Normal mucosa (NM) and primary tumor (PT) of 40 metastatic gastric adenocarcinomas patients who received second-line paclitaxel-ramucirumab (PR) were analyzed for mRNA expression of the following genes: *HK-1, HK-2*, *PKM-2*, *LDH-A*, and *GLUT-1*. Patients were categorized with PGP when at least a doubling of mRNA expression (PT vs. NM) in all glycolytic core enzymes (HK-1 or HK-2, PKM-2, LDH-A) was observed. PGP was also related to *TP53* mutational status.

**Results:**

Mean *LDH-A*, *HK-2*, *PKM-2* mRNA expression levels were significantly higher in PT compared with NM. 18 patients were classified as PGP, which was associated with significantly worse progression-free and overall survival times. No significant association was observed between PGP and clinical-pathologic features, including *TP53* positive mutational status, in 28 samples.

**Conclusions:**

Glycolytic proficiency may negatively affect survival outcomes of metastatic gastric cancer patients treated with PR systemic therapy. *TP53* mutational status alone does not seem to explain such a metabolic shift.

**Electronic supplementary material:**

The online version of this article (10.1007/s10120-020-01078-0) contains supplementary material, which is available to authorized users.

## Background

**Fig. 1:**
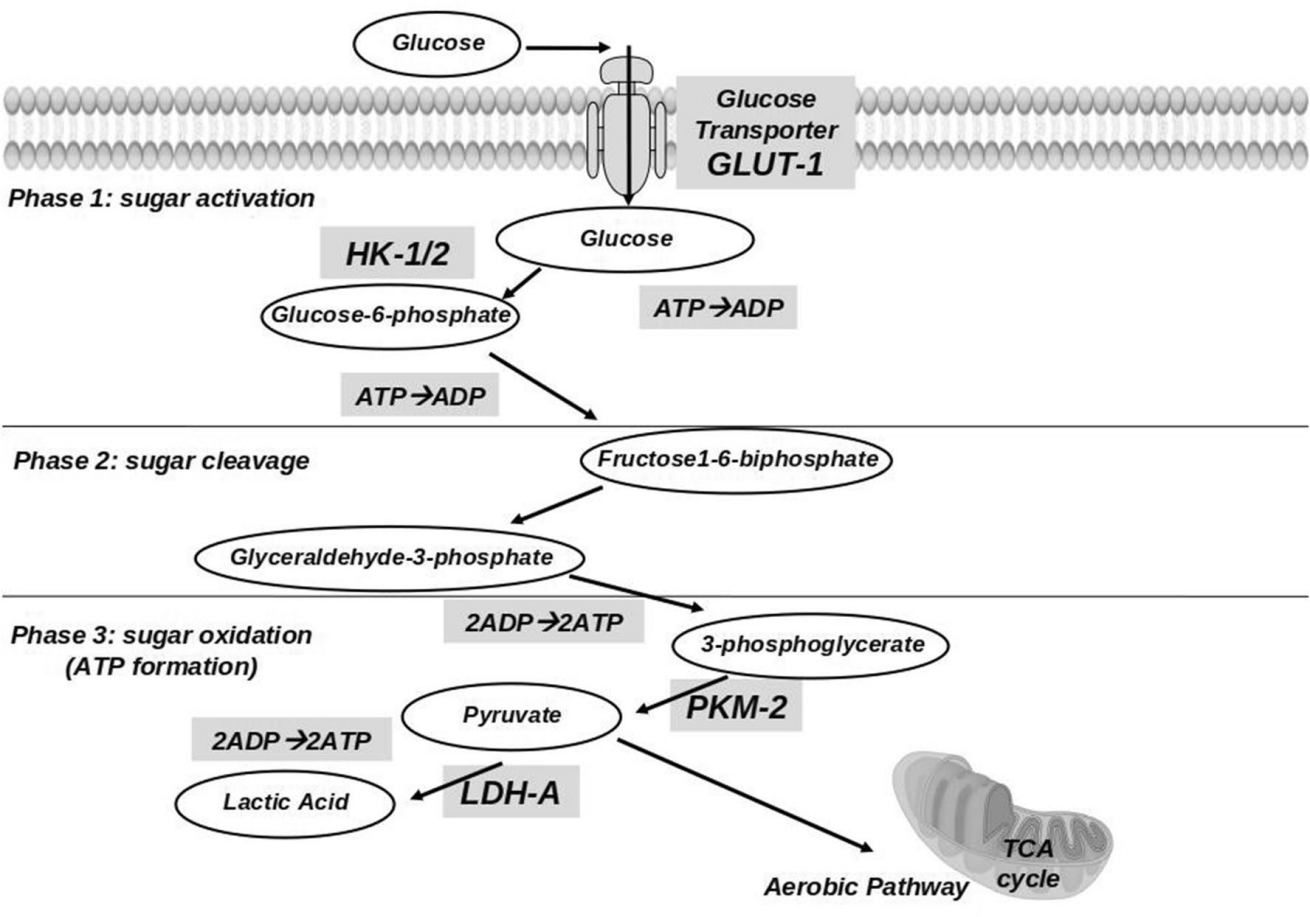
Schematic representation of the three main steps (sugar activation, cleavage and oxidation) in the glycolysis pathway. Glucose transport-1 (GLUT-1) mediates the internalization of glucose across the plasma membrane. Hexokinase (HK-1 and HK-2) transfer one phosphate group from ATP to glucose, yielding glucose-6-phosphate (G6P). G6P may be shunted into the non-oxidative arm of the pentose phosphate pathway (PPP), otherwise it is converted through the intermediate reaction of glycolysis to 3-phosphoglycerate. Pyruvate kinase (PKM-2) catalyzes the transfer of a phosphate group from 3-phosphoglycerate to ADP, to give pyruvate and ATP. In the presence of oxygen, cells completely oxidize most of that pyruvate in the mitochondria to CO_2_ during the process of oxidative phosphorylation in the tricarboxylic acid (TCA) cycle. When oxygen is limited, cells can redirect the pyruvate generated by glycolysis away from mitochondrial oxidative phosphorylation by generating lactate (anaerobic glycolysis). Lactate dehydrogenase isoform A (LDH-A) catalyzes the reversible conversion of pyruvate to lactate with the simultaneous oxidation of the cofactor NADH to NAD^+^. Warburg observed that cancer cells tend to convert most glucose to lactate regardless of whether oxygen is present (aerobic glycolysis)

For energy production under aerobic conditions, normal cells generally transform glucose into carbonic anhydride by means of oxidative phosphorylation. Conversely, glycolysis with ultimate production of lactate is predominant in invasive cancer cells, even in the presence of sufficient levels of oxygen [1]. Although the incomplete oxidation of glucose to lactate yields only 5% of the energy available from glucose, this apparently senseless waste of glucose actually constitutes a survival advantage in rapidly proliferating cells. In fact, it makes them insensitive to transient or permanent hypoxic conditions, it contributes to the production of nucleosides and amino acids, and it constitutes a very rapid way to produce energy [1, 2]. Furthermore, lactate is not just a waste product of this process; on the contrary, it promotes tumor invasion by favouring cell migration, angiogenesis, immune escape and radioresistance [3]. This metabolic shift is promoted by the over-expression of the key effectors of the glycolytic pathway [4, 5], including specific membrane glucose transporters (GLUT-1), and enzymes involved in the promotion of each single step of the glycolytic cascade (Fig. 1). The over-expressed enzymes themselves are subject to selection with some isoforms more frequently represented in tumor cells [4, 5].

In a previous study in metastatic colorectal cancer [6], we found mRNA tumor overexpression of *GLUT-1* and the glycolytic genes hexokinase 1 (*HK-1*) and *2 *(*HK-2*), pyruvate kinase isoform 2 (*PKM-2*) and lactate dehydrogenase isoform A (*LDH-A*). In the subset of patients treated with anti-angiogenic bevacizumab, the glycolytic profile showed signals of detrimental association with survival outcomes [6]. In fact, clones can be selected that have the ability to survive anti-angiogenic therapy‐induced hypoxia, and the selection of hypoxia ‐resistant clones can also be observed with VEGF (vascular epithelial growth factor) receptor-1 and -2 inhibition [7, 8]. These clones require fewer pro-angiogenic factors to promote their growth and proliferation and they possess phenotypic properties allowing them to overcome the lack of energy and nutrients supply [7, 8]. Most relevant, metabolic adaptation with a glycolytic shift may not be a simple prognostic biomarker [9, 10], but it may indicate innovative treatment strategies and novel drug targets in anti-cancer therapy [11–13].

This background prompted us to plan a novel study for evaluating the possible negative impact of the up-regulated glycolytic profile in patients exposed to anti-angiogenics. We focused on the paclitaxel-ramucirumab (PR) association for second-line therapy in metastatic gastric cancer. Ramucirumab is a recombinant human immunoglobulin G1 (IgG1) neutralizing monoclonal antibody specific for VEGF receptor-2 that prevents ligand binding and receptor-mediated pathway activation in endothelial cells. It is approved as a single agent, or in combination with paclitaxel, for the treatment of patients with advanced or metastatic gastric or gastroesophageal junction cancer with disease progression or after prior fluoropyrimidine or platinum-containing chemotherapy [14].

Interestingly, recent studies have revealed novel functions of the *TP53* tumor-suppressor gene including the regulation of glycolysis [15]. In pre-clinical models, the p53 protein has been shown to repress glycolysis through multiple mechanisms. In particular, p53 transcriptionally represses the expression of glucose transporters and it was found to down-regulate the HK-2 and PKM-2 glycolytic enzymes. Also, p53 induces the expression of TIGAR (*TP53*-induced glycolysis and apoptosis regulator), which decreases the intracellular concentrations of fructose-2,6-bisphosphate, and thus reduces glycolysis and diverts glucose catabolism to the pentose phosphate pathway [16, 17]. The *TP53* gene is frequently mutated in gastric adenocarcinomas, and unlike many other tumor suppressors, the majority of *TP53* mutations are missense, which usually leads to the production of the full-length mutant protein [18]. Also, it has been well-documented that some mutant p53 proteins not only lose the tumor-suppressive function, but they gain new oncogenic functions as a result of the so-called “gain-of-function” *TP53* mutations [18, 19]. In this study, we also devoted an ancillary analysis to *TP53* mutational status to evaluate whether signals of p53 regulation of the glycolytic shift are detectable in vivo.

## Methods

Italian institutions involved in the RAMos retrospective study [20] were asked to participate in the present study. To evaluate the results of a translational analysis in a homogeneous population of patients, this retrospective investigation focused on patients treated with the combination of ramucirumab and paclitaxel only. Therefore, patients were required to be treated with ramucirumab 8 mg/kg on days 1 and 15, with paclitaxel 80 mg/m^2^ on days 1, 8 and 15, and both intravenously every 28 days. Availability of paired tissues of the primary tumor (PT) and normal mucosa (NM) was required for study inclusion. To characterize the glycolytic shift in cancer cells, mRNA over-expression of key enzymes in the three main phases of the glycolytic pathway were studied (Fig. 1). The relationship between levels of the mRNA and survival outcomes was assessed. All patient information and pathology material were collected under a protocol approved by the Regional Ethical Committee. Patients were asked to provide additional written informed consent (see supplementary information file).

### Samples and nucleic acids extraction

Four to six 10-μm sections from formalin-fixed, paraffin-embedded (FFPE) specimens were obtained from PT and matched NM. The sample preparation protocol expressly indicated the acquisition of NM samples from surgical or biopsy blocks with accurate identification of healthy gastric mucosa. These sections had to be distinct from those prepared for tumor sampling thus excluding proximity to tumor infiltration. Before cutting sections for total nucleic acids isolation, an additional slide was prepared for hematoxylin–eosin staining and the pathologists identified representative areas with an almost complete representation of tumor infiltration. For each enrolled patient, total DNA and RNA were extracted from PT and matched NM. Both tissues were micro-dissected and placed in a 1.5 ml reaction tube containing 1 ml xylene to remove paraffin. DNA and RNA were extracted using the RecoverAll™ Multi-Sample RNA/DNA Isolation Workflow (Invitrogen™, CA, USA) according to the manufacturer's instructions. DNA and RNA concentration and purity were measured using the NanoDrop 1000 spectrophotometer (Nanodrop Technologies, Rockland, DE, USA).

### cDNA synthesis and quantitative real-time PCR (RT-qPCR)

The SuperScript^TM^VILO™ cDNA Synthesis kit (Invitrogen™, CA, USA) was used to generate first-strand cDNAs from 1 μg of total RNA according to the manufacturer's instructions. The products were diluted to obtain a final concentration of 10 ng/μl of reverse-transcribed mRNA. Quantitative real-time PCR (RT-qPCR) was performed to analyze the mRNA expression levels of the five candidate genes (*HK-1, HK-2, PKM-2, LDH-A, and GLUT-1*) and two reference genes (*B2M* and *GUSB*), as previously reported [6]. Briefly, RT-qPCR was carried out using TaqMan Gene Expression Assay and TaqMan Gene Expression Mastermix (Applied Biosystems, Foster City, CA, USA) following the manufacturer’s protocol. All reactions were performed in triplicate and each PCR run included a no-template control. The RT-qPCR Ct values (the average value of the triplicates) were converted in Cy0 by a tool for accurate and precise quantification [21] and the relative mRNA expression of each target was calculated as ΔCy0 = Cy0_(target gene)_—Cy0_(reference gene)_. In this analysis, a higher mRNA expression level corresponds to a smaller ΔCy0 value.

Subsequently, the 2^−ΔΔCy0^ method was used to express the n-fold differential expression (fold change) of each candidate gene between the tumor sample and the normal counterpart. Fold change ≥ 2 indicates a doubling in the mRNA content and it was adopted as a threshold for differential RNA expression in microarray and drug induction studies [22–25].

### Amplicons library preparation and next-generation sequencing (NGS) for TP53 analysis

A custom panel (IAD_119861) including the *TP53* coding and the relative UTR regions was designed using the Ion AmpliSeq™ Designer software (Thermo Fisher, Foster City, CA). The panel was made up of 35 amplicons and ensured 82% of gene coverage. The Ion AmpliSeq Library Kit Plus was used to prepare the libraries according to the manufacturer’s instructions. Libraries were generated using 40 ng of tumor DNA and indexed using the Ion Xpress Barcode Adapter Kit. Library purification was carried out using the AMPure™ XP Reagent (Beckman Coulture, CA, USA) on the DynaMag™-2 Magnet. Qubit™ 4 Fluorometer (Invitrogen™,, CA, USA) was used to quantify amplicons libraries. After dilution of all samples at 100 pM, libraries were pooled for emulsion PCR on the Ion OneTouch™ 2 instrument, using the Ion S5™ Template OT2 kit, according to the manufacturer’s instructions. The Ion Sphere™ Particles were enriched using the Ion OneTouch™ Enrichment System and the template was sequenced on the Ion Torrent S5 platform using the Ion 540™ Chip following the manufacturer’s instruction. All of these instruments and reagents were supplied by Thermo Fisher (Foster City, CA). Read alignment was performed using hg19 (GRCh37) as the reference genome.

Variant call files were generated by the Variant Caller v.5 plugin pre-installed in the Torrent Suite and analyzed with the Ion Reporter™ software (Thermo Fisher, Foster City, CA). BAM files were also manually checked on IGV (Integrative Genomics Viewer) [26]. Mutations were categorized as “disruptive” (*TP53*_D_) or “non-disruptive” (*TP53*_ND_) according to the classification of Poeta et al. [27]. Mutations were also classified as “gain-of-function” (*TP53*_GOF_) if reported in current databases from the review of available studies in which a clear gain-of-function effect was shown [19].

### Statistical analysis

mRNA expression levels were reported as ΔCy0 values with means and standard deviations; group differences were compared using two-sample t- and Wilcoxon tests. Significant associations for each gene were required to be detectable with both reference genes. Contingency tables were analyzed by the Fisher’s exact test. Statistical significance was defined as *p* < 0.05.

For the purpose of clinical associations, fold change results produced by the 2^−ΔΔCy0^ method were adopted. Cases were defined as having a positive glycolytic profile (PGP) when fold changes ≥ 2 (PT vs. NM) were present in all glycolytic core genes: *HK-1 or HK-2, PKM-2, LDH-A, and GLUT-1*. The remaining cases were categorized as having a negative glycolytic profile (NGP).

The primary end-point was overall survival (OS), which was compared between PGP and NGP groups to assess the possible clinical impact of glycolytic proficiency. OS was calculated from the date of the first cycle of second-line PR therapy to the earliest of date of death or last follow-up. Progression-free survival (PFS) was the secondary end-point, defined as the time from the date of the first cycle of second-line PR therapy to the earlier of disease progression or death, or last follow-up.

The Kaplan–Meier method was used to estimate survival curves and the log-rank test was used to compare survival times between PGP and NGP groups. A multivariable Cox proportional hazards model was then used to adjust for clinical and pathologic features. Two-sided *p* values 95% confidence intervals (CI) were reported. A *p *value < 0.05 was considered statistically significant.

Statistical analyses were performed using MedCalc for Windows, version 15.0 (MedCalc Software, Ostend, Belgium).

## Results

Forty consecutive patients who underwent PR second-line systemic therapy and had paired archival tissue samples of the PT and matched NM were included from eight Italian institutions. All patients received first-line chemotherapy with a platinum derivate (cisplatin or oxaliplatin) plus fluoropyrimidines. In the second-line setting, patients underwent PR between September 2015 and September 2018 (Table 1). The results of second-line therapy in this cohort of patients parallel findings in the RAMos study [20]. The overall response rate was 17.5%, with 7 partial responses in the 40 patients. In the whole group, the median OS was 7.8 months (95% CI 4.5 to 8.6 months) and the median PFS was 3.8 months (95% CI 3.2 to 4.6 months).

**Table 1:** Characteristics and distribution of the 40 patients according to the glycolytic profile

Variable	Number of patients (%)			
	Total	PGP	NGP	*p* value
Age				
> 70 years	29 (72.5)	11 (61)	18 (82)	0.7
< 70 years	11 (27.5)	7 (39)	4 (18)	
Gender				
Male	18 (45)	7 (39)	11 (50)	0.5
Female	22 (55)	11 (61)	11 (50)	
PFS1 time				
> 6 months	26 (65)	13 (72)	13 (59)	0.5
< 6 months	14 (35)	5 (28)	9 (41)	
Number of metastatic sites				
1	21 (52.5)	6 (33)	15 (68)	0.05
> 2	19 (47.5)	12 (67)	7 (32)	
Peritoneum involvement				
Positive	21 (52.5)	10 (55)	11 (50)	0.7
Negative	19 (47.5)	8 (45)	11 (50)	
ECOG PS				
0	22 (55)	10 (55)	12 (54.5)	0.5
1–2	18 (45)	8 (45)	10 (45.5)	
Lauren’s histology				
Intestinal	26 (65)	14 (78)	12 (54.5) 0.9	
Diffuse	14 (45)	4 (22)	10 (45.5)	
Grading				
1–2	18 (45)	10 (55)	8 (36)	0.3
3	22 (55)	8 (45)	14 (64)	
Primary tumor resected				
Yes	18 (45)	7 (39)	11 (50)	0.1
No	22 (55)	11 (61)	11 (50)	
Primary tumor site				
Cardia	9 (22.5)	5 (28)	4 (18)	0.7
Non-cardia	21 (77.5)	13(72)	18 (82)	

*PGP* positive glycolytic profile, *NGP* negative glycolytic profile, *PFS1* progression-free survival to first-line chemotherapy, *ECOG PS* Eastern Cooperative Group Performance Status

### Expression analyses

**Fig. 2:**
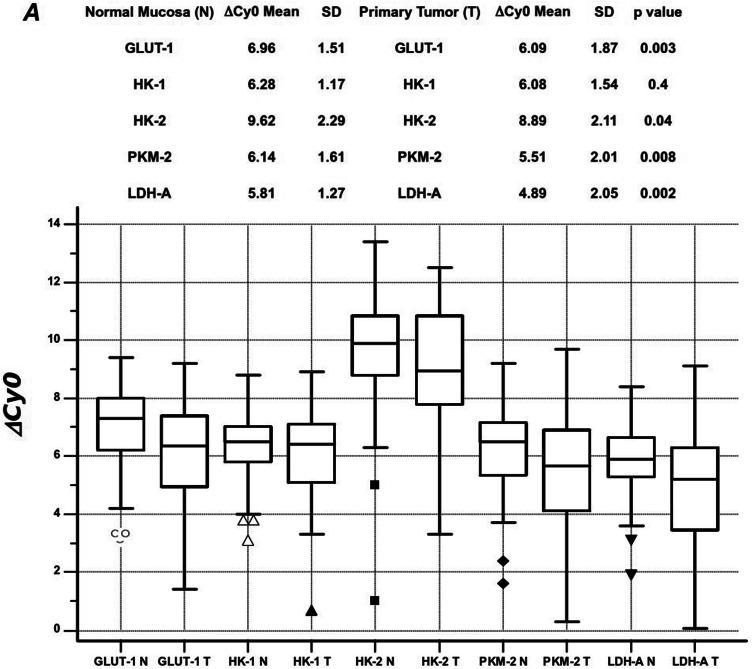
Box plot with standard deviation (SD) bars showing mRNA expression levels of the candidate genes in the primary tumor (T) and normal mucosa (N). Data are presented as ΔCy0 values: the smaller the ΔCy0 value, the higher the expression

As shown in Fig. 2, statistically significant differences in mRNA expression levels were detected comparing ΔCy0 values between PT tissues and matched NM for *HK-2, PKM-2, GLUT-1* and *LDH-A*.

**Fig. 3:**
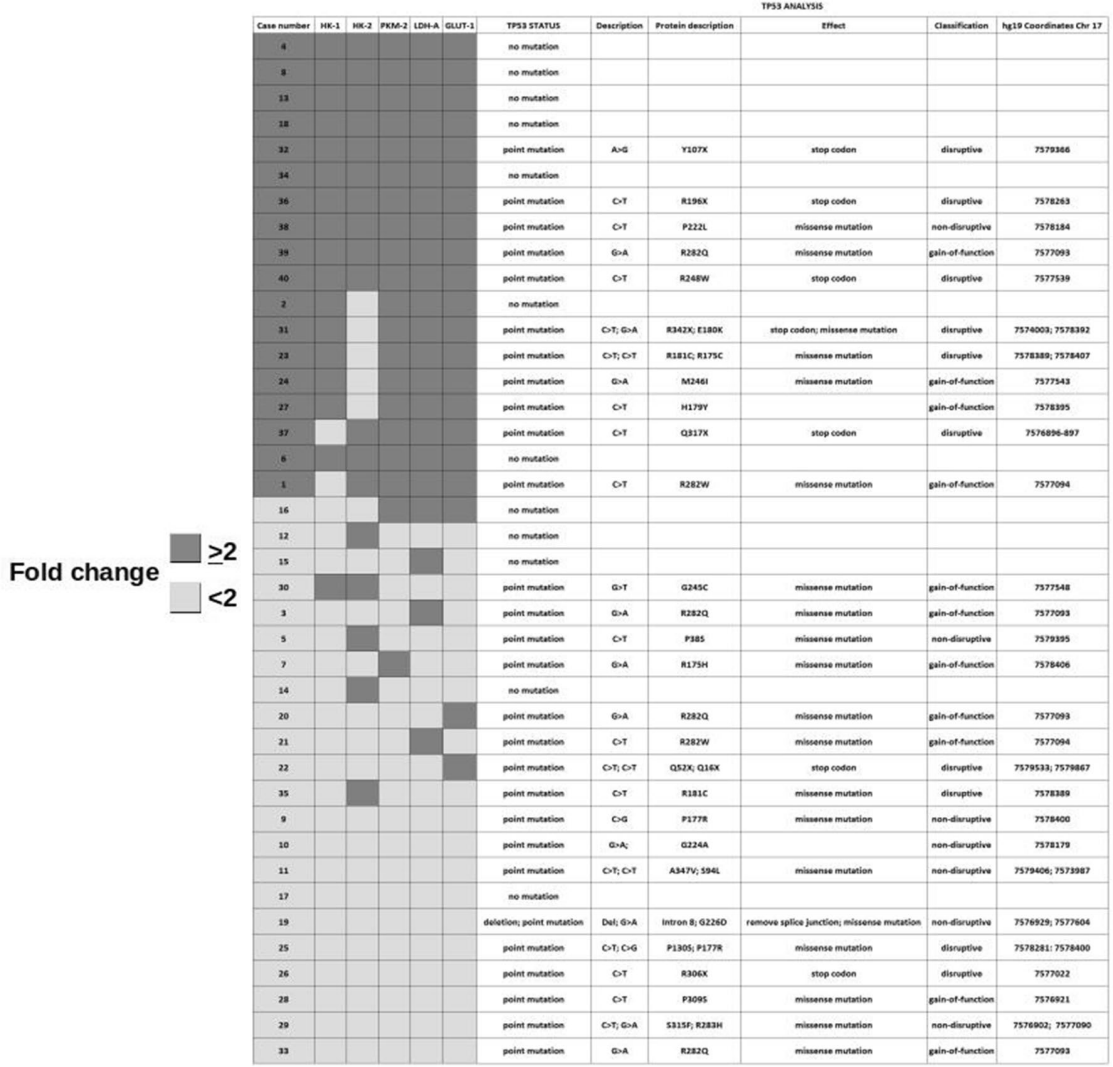
Results of tumor profiling according to fold-change mRNA analysis. Cases were categorized as positive glycolytic profile (PGP) when fold-change ≥ 2 simultaneously occurred in *HK-1* or *HK-2*, *PKM-2*, *LDH-A* (dark grey squares). White squares denote the remaining cases with a negative glycolytic profile (NGP) because fold-change < 2 or fold- change ≥ 2 in only one or two mRNA

The ranking of fold change analysis is reported in Fig. 3. Fold change ≥ 2 was detected in 19 cases for *GLUT-1*, in 19 cases for *LDH-A*, in 19 cases for *PKM-2*, in 13 cases for *HK-2* and in 16 cases for *HK-1*. Eighteen cases showed synchronous fold change ≥ 2 in one of the two *hexokinases* (*HK-2* or *HK-1*), *PKM-2, LDH-A*, and they composed the PGP group. Notably, all the PGP cases showed also fold change ≥ 2 for *GLUT-1*. The remaining 22 cases composed the NGP group. Intriguingly, a clear-cut distribution of fold change ≥ 2 expression levels in the four analyzed target genes seems to be present. In fact, except for case number 16 and 30, NGP group cases showed none or a single mRNA fold change ≥ 2.

As shown in Table 1, the distribution of clinical and pathologic features of the 40 patients according to PGP and NGP status did not show statistically significant associations. A borderline *p *value (*p* = 0.05) was observed between PGP and NGP groups for the number of metastatic sites, with a numerically greater prevalence of PGP patients having more extensive metastatic disease.

PGP status did not differentiate treatment responses to second-line therapy (*p* > 0.05). There were 2 partial response in the PGP group and 5 in the NGP group. Four patients had stable disease in the PGP group and 10 in the NGP group. Disease progression occurred in 12 patients in the PGP group and in 7 patients in the NGP group. The number of patients with partial response and stable disease in the disease-control rate (DCR) was statistically significantly different between the two groups with DCR in 15 and 6 patients in the NGP and PGP groups, respectively (*p* = 0.03).

### Expression analysis and TP53 status

*TP53* mutations in the coding region were found in 28 patients (70%). All detected *TP53* mutations were missense mutations described in the IARC database. Their characteristics are reported in Fig. 3, together with the distribution of cases according to glycolytic status. Eleven carriers of a *TP53* mutation were in the PGP group and 17 were in the NGP group. Seven patients without *TP53* mutations were in the PGP group and 5 in the NGP group. The association between *TP53* mutation and glycolytic status was not statistically significant (*p* = 0.20). The *TP53* missense mutations were classified as *TP53*_ND_ in 7 cases, *TP53*_D_ in 10 cases and *TP53*_GOF_ in 11 cases. In particular, all but one of the *TP53*_ND_ mutations occurred in the NGP group. Six of the 10 *TP53*_D_ mutations were in the PGP group and 7 of the 11 *TP53*_GOF_ mutations were the NGP group (*p* = 0.1).

### Survival analysis

**Fig. 4:**
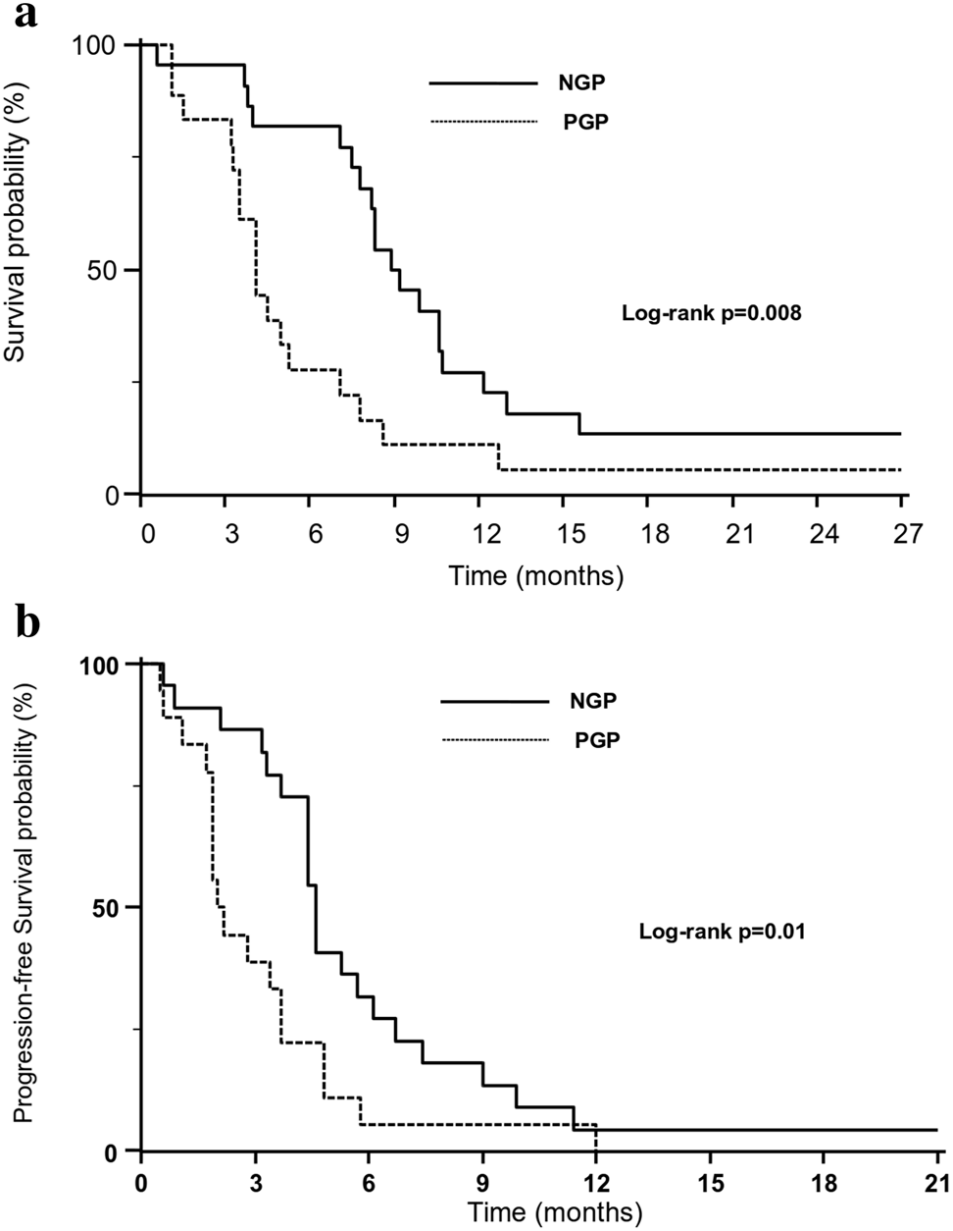
Results of survival analyses by PGP and NGP status in the 40 patients. Kaplan-Meyer survival curves of overall survival (Panel A) and progression-free survival (Panel B) to second-line paclitaxel-ramucirumab

In the OS analysis of second-line PR systemic therapy (see Fig. 4, Panel A), patients with a NGP showed statistically significant better survival than those with PGP: median OS of 8.9 months (95% CI 7.8–10.7 months) vs. median OS of 4.1 months (95% CI 3.5–5.3 months), respectively (*p* = 0.008). Similarly, glycolytic status showed a statistically significant impact on PFS (Fig. 4, panel B). Median PFS in NGP patients was 4.9 months (95% CI 4.4–6.1 months) and median PFS in PGP patients was 2.0 months (95% CI 1.9–3.7 months) (*p* = 0.02).

The results of the multivariable Cox regression analysis are shown in Table 2. The glycolytic profile retained independent associations with PFS and OS, after controlling for other prognostic factors. In the OS analysis, adverse outcome was associated with PGP status, ECOG performance status 1–2, peritoneum involvement and the presence of the primary gastric tumor.

**Table 2:** Results of the multivariate model analysis

	Overall survival		Progression-free Survival	
Variable	HR (95% confidence interval)	*p *value	HR (95% confidence interval)	*p *value
Age				
> 70 years vs. < 70 years	0.51 (0.21–1.16)	0.1	0.64 (0.29–1.39)	0.2
Gender				
Female vs, Male	0.53 (0.19–1.46	0.5	0.48 (0.53–3.78)	0.4
First PFS time				
< 6 months vs, > 6 months	2.41 (0.92–6.33	0.07	1.38 (0.60–3.16)	0.4
Number of metastatic sites				
> 2 vs. 1	1.27 (0.55–2.96)	0.5	1.32 (0.59–2.95)	0.5
Peritoneum involvement				
Positive vs. Negative	2.94 (1.21–7.13)	0.01	1.42 (0.60–3.35)	0.4
ECOG PS				
1–2 vs: 0	2.56 (1.18–5.56)	0.01	1.83 (0.88–3.79)	0.1
Lauren’s histology				
Intestinal vs. Diffuse	0.46 (0.16–1.33)	0.4	0.64 (0.26–1.54)	0.3
Grading				
3 vs.1–2	1.43 (0.46–4.43)	0.5	1.58 (0.48–5.10)	0.4
Primary tumor resected				
No vs. Yes	3.11 (1.03–9.38)	0.04	3.39 (1.05–10.09)	0.04
Primary tumor site				
Cardia vs. non-cardia	2.55 (0.79–8.26)	0.1	1.81 (0.66–5.39)	0.4
Glycolytic status				
PGP vs. NGP	2.57 (1.17–5.63)	0.01	2.49 (1.16–5.38)	0.01

*HR* hazard ratio, *PGP* positive glycolytic profile, *NGP* negative glycolytic profile, *PFS* progression-free survival, *ECOG PS* Eastern Cooperative Group Performance Status

## Discussion

Even after a decade from the FDA approval of the first anti-VEGF drug bevacizumab, resistance to anti-VEGF therapy remains a challenge in the treatment of cancer patients. Mechanisms of resistance are described as being intrinsic (preexisting) or adaptive (acquired), and they may explain why some tumors do not respond from the outset and why others progress after initial shrinkage. Redundancy of pro-angiogenic growth factors and activation of alternative angiogenic pathways have been investigated and considered as the prevalent mechanisms of resistance to anti-angiogenic compounds in cancer therapy [8, 9]. In recent years, many pre-clinical and translational studies indicated that metabolic reprogramming with adaptive escape in response to a hypoxic tumor microenvironment may offer a novel and intriguing opportunity for explaining the failure of anti-angiogenic treatments in solid tumors. In particular, glycolysis is an essential metabolic pathway in the hypoxic adaptation for survival and tumor progression. In this perspective, tumors may develop early and/or late resistance to anti-angiogenic agents when clones are more equipped for prompt and redundant metabolic changes (i.e., glycolytic shift) in a therapeutically induced hypoxic environment [7, 9].

In the past few years, translational clinical studies in cancer patients have addressed the putative clinical impact of glycolysis-related proteins and factors on prognosis, and there is mounting evidence that these features negatively affect survival outcomes [28]. The gastric cancer setting was analyzed in some of these studies. Findings showed significant associations between poor prognosis and more advanced stage disease or adverse histological features with up-regulated expression of glucose transporters [29–31] hexokinases [32–34] pyruvate kinases [34, 35], other enzymes involved in energy metabolism [36, 37], and lactate dehydrogenase [38–40]. To the best of our knowledge, this is the first study that analyzes a homogenous population of metastatic gastric cancer patients treated with a regimen that includes an anti-angiogenic compound. Moreover, we attempted an approach with multiple genes to determine a PGP rather than a single-component analysis.

A starting point for discussion is the characterization of the population of gastric cancer patients whose tumors displayed the positive glycolytic profile. Given the relatively early interest in the clinical impact of cancer metabolic features, there is a lack of standardized criteria and almost all studies investigated single glycolysis-related factors with different methods [28]. We formulated an approach which combined the biochemical principle of the glycolytic cascade in its main enzymatic steps [41], together with a sensitive threshold for mRNA expression in vivo [23, 25]. Fold-change is a very intuitive method to identify differentially expressed genes and it quantifies the change of expression levels. In fold-change analyses, 1.5 or 2 is often used as the cut-off to choose differentially expressed genes. The contemporary up-regulation of three key glycolytic enzymes coupled with fold-change ≥ 2 in mRNA expression in cancer tissues compared with their normal counterpart is a fairly strict criterion to label a PGP case. In a comparative analysis between tissues, the quality of sampling is mandatory for reducing the risk of biases. For example, the result of a NGP status could be a false-negative if sampling was made in deceptively healthy NM areas. In our study, pre-specified procedures for sampling and the involvement of expert pathologists in the selection of tissues should have minimized this risk. The observed differences in mRNA expression levels of target genes between NM and PT tissues would suggest an effective procedure to seize the presence of the glycolytic shift. We cannot rule out that an expression level analysis of each target gene in PT or NM tissues could be also predictive of clinical outcomes. However, to our opinion, this approach would be less informative on a global glycolytic shift and it could be done after identifying a cut-off level for each tested gene. We acknowledge that our criteria necessitate replication in independent studies and additional settings. Hopefully, they could lay the groundwork for a standardized determination of a glycolytic profile in translational cancer studies.

In the global analysis of mRNA levels between PT tissues and NM, enhanced expression of *GLUT-1*, *PKM-2*, *LDH-A* and *HK-2* was found. Except for *HK-1*, these data parallel our findings in colorectal cancer [6]. The variable result of *HK-1* expression analysis in cancer tissues and the lack of global up-regulation in this study is not surprising. *HK-2* was found to be overexpressed more than *HK-1* in several cancer types compared with their normal counterpart [42]. The four hexokinase isoenzymes (HK-1, HK-2, HK-3, and glucokinase) are structurally similar, but only HK-1 and HK-2 are functionally similar. Since hexokinase serves as the gateway through which glucose enters the alternative metabolic pathway, *HK-1* is redundant to the primary catalytic role of HK-2 to ensure the cancer cell a constant glycolytic flux [43].

According to our criteria, 18 (45%) primary gastric adenocarcinomas were categorized as having a positive glycolytic profile and, therefore, displaying an intrinsic capability of metabolic adaptation in an unfavorable, hypoxic microenvironment. In fact, the PR combination is not a “pure” anti-angiogenic treatment, but it has been demonstrated that even paclitaxel exploits anti-angiogenic mechanisms of action, especially in fractionated regimens [44]. These features may explain why the exposure of these patients to an anti-angiogenic therapy with ramucirumab and paclitaxel produced significantly shorter OS and PFS than patients with a negative glycolytic profile. More than providing novel prognostic information, mediators of an up-regulated glycolytic status may represent the target of novel compounds with tumor metabolism interference activity [45]. Inhibitors of glucose transporters, PKM-2 and LDH-A, attenuate aerobic glycolysis and tumor proliferation with the potential therapeutic role [46], alone or in combination with anti-angiogenic [47] and immune checkpoint [48] therapies.

Unlike our previous analysis in colorectal cancer [6], we did not evaluate *RAS* mutations. This choice was made considering the low frequency of *RAS* mutations in gastric cancer (roughly 1–10%) and the presence instead of *RAS* amplification [49]. Notably, RAS mutations and *RAS* amplification may display different oncogenic effects in molecular pathways [49], thus, making it even more difficult to interpret the role of oncogenic *RAS* in gastric adenocarcinoma*.* Conversely, we attempted an exploration between glycolytic status with PGP/NGP categories and *TP53* mutations. This analysis was supported by high frequency of *TP53* mutations in gastric adenocarcinomas [18, 19] and the increasing amount of pre-clinical and experimental data, which support a major role of the tumor suppressor gene in orchestrating the glycolytic capability of cancer cells [15, 17]. *TP53* mutations were almost equally distributed between PGP and NGP, without any significant association even after considering their sub-classification into disruptive, non-disruptive and gain-of-function.Proof of *TP53* driving the Warburg effect mainly derive from pre-clinical studies in cellular and murine models [15, 17]. It is likely that glycolytic properties of cancer cells in vivo undergo modulation from multiple effectors [50]. Therefore, the putative influence of p53 perturbation is diluted among the several factors that may impact the glycolysis capabilities of cancer cells.

Limitations of this study are its retrospective nature and small sample size. However, our sophisticated analysis of glycolysis profiling in gastric adenocarcinomas, which required primary tumor tissues and the normal mucosa, lays a foundation for future study. This is especially important since no affordable predictive marker of response and survival under the ramucirumab-paclitaxel regimen has been identified [51] and second-line therapy is offered to all patients who may benefit according to clinical criteria.

In conclusion, glycolytic proficiency was found to be associated with adverse survival outcomes of metastatic gastric cancer patients treated with PR systemic therapy. *TP53* mutational status alone does not seem to explain such a metabolic shift. Further investigation is needed to confirm our findings that would promote the development of novel therapeutic strategies against cancer metabolism.

## Electronic supplementary material

Below is the link to the electronic supplementary material.

Supplementary file1 (PDF 341 kb)
