# Supplementary material for: Glycolytic competence in gastric adenocarcinomas negatively impacts survival outcomes of patients treated with salvage paclitaxel-ramucirumab
Source: Gastric Cancer. 2020 May 5;23(6):1064–74. doi: 10.1007/s10120-020-01078-0 (PMC7567716; doi:10.1007/s10120-020-01078-0)
Supplement: Supplementary file 1 — Supplementary file1 (PDF 341 kb) [file 10120_2020_1078_MOESM1_ESM.pdf]

CerM
comitato etico regione marche

*Comitato Etico Regionale delle Marche*
*c/o Azienda Ospedaliero-Universitaria Ospedali Riuniti*
*Via Conca, 71 - 60126 Torrette di Ancona*
*Presidente Prof. Paolo Pelaia*

**Seduta del 21 luglio 2016**

Il Comitato Etico Regionale delle Marche (CERM), istituito con delibere n. 571 del 31/10/2014 e n. 1 del 8/1/2015 in esecuzione della DGRM n. 1104 del 29 settembre 2014, è organizzato ed opera secondo quanto indicato dal D.M. 189/2012 e per le sue decisioni ed attività relative alle sperimentazioni cliniche fa riferimento, alle norme di "Good Clinical Practice" (ICH-GCP) nella versione più recente.

Il Comitato Etico nella seduta del **giorno 21 luglio 2016** ha preso in considerazione il protocollo della sperimentazione dal titolo "Analisi dell'espressione di enzimi correlati alla glicolisi aerobia nel carcinoma gastrico e possibile impatto clinico sul trattamento anti-angiogenetico con ramucirumab + chemioterapia di seconda linea nella malattia metastatica"-SMAC gastric (sponsor/promotore: U.O.C. ONCOLOGIA, AORMN, P.O. San Salvatore di Pesaro - Stabilimento Muraglia Via Lombroso 1 – 61122 Pesaro) proposto dal Dott. Francesco Graziano, U.O.C. Oncologia, Presidio Ospedaliero San Salvatore di Pesaro - Stabilimento Muraglia Via Lombroso 1 – 61122 Pesaro.

Dopo aver esaminato:

**Elenco documenti inviati a supporto della lettera di richiesta di valutazione datata**
**Allegato_n._6_studio osservazionale, Allegato_n._11_conflitto interessi del dr.Francesco Graziano, Allegato_n._8_natura osservazionale dello studio centro coordinatore, Allegato_n._7_ studio osservazionale no_profit, Curriculum vitae recente del dr.Francesco Graziano firmato e datato, Sinossi e protocollo dello studio versione 01 del 04/05/16, elenco centri partecipanti versione 01 del 04/05/16, Foglio informativo e consenso informato versione 01 del 04/05/16, lettera di intenti del 04/05/16**

Valutata l'idoneità della struttura ove sarà effettata la ricerca e l'idoneità dello sperimentatore all'effettuazione dello specifico studio, **il Comitato esprime PARERE FAVOREVOLE alla sperimentazione in esame.**

Ogni cambiamento del protocollo di studio dovrà essere trasmesso al Comitato Etico.

Si richiede che questo Comitato Etico venga informato dell'inizio della sperimentazione e della sua conclusione o eventuale interruzione. Inoltre dovrà essere informato di ogni successivo emendamento al protocollo e degli eventi avversi, seri o inattesi insorti nel corso dello studio, che potrebbero influire sulla sicurezza dei soggetti o sul proseguimento dello studio.

**Protocollo N. 2016-0374 MN**

**IL PRESIDENTE**
**Prof. Paolo Pelaia**

COMITATO ETICO REGIONALE
DELLE MARCHE (C.E.R.M)
c/o Azienda Ospedaliero Universitaria
Ospedali Riuniti
Via Conca, 71 - 60126 Torrette - ANCONA

## Elenco presenze della Riunione del Comitato Etico Regionale delle Marche (C.E.R.M.) del 21 luglio 2016

| NOMINATIVO COMPONENTE | CARICA | ENTE DI RIFERIMENTO | RUOLO | PRESENZA SI/NO |
|---|---|---|---|---|
| PROF. PAOLO PELAIA | PRESIDENTE CERM | AOU OSP.RIUNTI | CLINICO | SI |
| PROF. SALVATORE AMOROSO | RESP. STS | AOU OSP.RIUNTI | FARMACOLOGO | NO |
| DOTT. ROBERTO ANTONICELLI | | INRCA ANCONA | CLINICO | SI |
| DOTT. VINCENZO BERDINI | | ESTERNO | MMGT | SI |
| DOTT. STEFANO BIANCHI | In relazione agli studi svolti presso Azienda Ospedaliera Marche Nord | A.O. MARCHE NORD | DIRETTORE SANITARIO (SOSTITUTO PERMANENTE) | NO |
| DOTT. GIUSEPPE BRAICO | | ESTERNO | PEDIATRA LIBERA SCELTA | SI |
| PROF. FLAVIA CARLE | | ESTERNO (UNIVPM) | BIOSTATISTICO | NO |
| DOTT. ANTONIO CHERUBINI | | INRCA ANCONA | CLINICO | SI |
| DOTT.SSA VALENTINA COLA | | AOU OSP.RIUNITI | ESPERTO DISPOSITIVI MEDICI | SI |
| ING. GIANCARLO CONTI | In relazione all'area medico-chirurgia con dispositivo medico | A.O. MARCHE NORD | INGEGNERE CLINICO | NO |
| DOTT. CARLO COSTANTINI | | ASUR | CLINICO | NO |
| PROF. MARCELLO D'ERRICO | PRESIDENTE VICARIO | AOU OSP.RIUNITI | CLINICO | SI |
| DOTT. PIERO GALIENI | | ASUR | CLINICO | SI |
| DOTT. MICHELE GENTILI | | ASUR | FARMACISTA SSR | NO |
| DOTT. MICHELE GIUA | | ESTERNO | RAPPRESENTANTE VOLONTARIATO O DELL'ASSOCIAZIONISMO DI TUTELA DEI PAZIENTI | NO |
| DOTT. COSTANTINO GOBBI | | ESTERNO | PEDIATRA LIBERA SCELTA | NO |
| DOTT. VINCENZO LARICCIA | | ESTERNO (UNIVPM) | BIOTECNOLOGO | SI |
| DOTT.SSA FABRIZIA LATTANZIO | In relazione agli studi svolti presso INRCA | INRCA ANCONA | DIRETTORE SCIENTIFICO | NO |
| DOTT. ALBERTO DEALES | In relazione agli studi svolti presso INRCA | INRCA ANCONA | DIRETTORE SANITARIO | NO |
| DOTT.SSA STEFANIA MAGGI | In relazione a studi con nuove procedure tecniche, diagnostiche e terapeutiche, invasive e semi invasive | A.O. OSPEDALI RIUNITI | CLINICO | NO |
| DOTT. MASSIMILIANO MARINELLI | | ESTERNO | BIOETICISTA | NO |
| DOTT. PAOLO MARINELLI | | INRCA ANCONA | RAPPRESENTANTE AREE SANITARIE | SI |
| DOTT. ANDREA MARINOZZI | | A.O. OSPEDALI RIUNITI | FARMACISTA SSR | SI |
| DOTT. VINCENZO MASSETTI | | ESTERNO | RAPPRESENTANTE VOLONTARIATO O DELL'ASSOCIAZIONISMO DI TUTELA DEI PAZIENTI | SI |
| DOTT. RODOLFO MATTIOLI | | A.O. MARCHE NORD | CLINICO | SI |
| PROF SSA LAURA MAZZANTI | In relazione a studi di prodotti alimentari | ESTERNO (UNIVPM) | ESPERTO IN NUTRIZIONE | SI |
| DOTT. FRANCESCO PELLEGRINI | | ASUR MARCHE | CLINICO | SI |
| DOTT. FRANCESCO PAOLO PERRI | | ASUR MARCHE | PEDIATRA DI LIBERA SCELTA | SI |
| DOTT. GIANLUCA SERAFINI | In relazione a studi svolti presso A.O. Ospedali Riuniti | A.O. OSPEDALI RIUNITI | DIRETTORE SANITARIO (SOSTITUTO PERMANENTE) | NO |
| DOTT. PAOLO SIGNORE | | ESTERNO | MMGT | SI |
| DOTT.SSA ROSA RITA SILVA | VICE PRESIDENTE | ASUR MARCHE | CLINICO | SI |
| DOTT.SSA NADIA STORTI | In relazione a studi svolti presso ASUR Marche | ASUR MARCHE | DIRETTORE SANITARIO | NO |
| PROF. ADRIANO TAGLIABRACCI | | A.O. OSPEDALI RIUNITI | MEDICO LEGALE | SI |
| DOTT. MARCELLO TAVIO | | A.O. OSPEDALI RIUNITI | CLINICO | SI |
| DOTT.SSA GIADA TORTORA | In relazione studi di genetica | ESTERNO | ESPERTO IN GENETICA | SI |
| SIG.RA CONCETTA TRAPE' | | ESTERNO | RAPPRESENTANTE VOLONTARIATO O DELL'ASSOCIAZIONISMO DI TUTELA DEI PAZIENTI | NO |

**Componenti del CERM presenti che non hanno partecipato alla votazione:**

COMITATO ETICO REGIONALE
DELLE MARCHE (C.E.R.M)
c/o Azienda Ospedaliero Universitaria
Ospedali Riuniti
Via Conca, 71 - 60126 Torrette - ANCONA
